# Detection of Melanoma Metastases in Resected Human Lymph Nodes by Noninvasive Multispectral Photoacoustic Imaging

**DOI:** 10.1155/2014/163652

**Published:** 2014-06-16

**Authors:** Gerrit Cornelis Langhout, Diederik Johannes Grootendorst, Omgo Edo Nieweg, Michel Wilhelmus Jacobus Maria Wouters, Jos Alexander van der Hage, Jithin Jose, Hester van Boven, Wiendelt Steenbergen, Srirang Manohar, Theodoor Jacques Marie Ruers

**Affiliations:** ^1^Department of Surgery, The Netherlands Cancer Institute, Antoni van Leeuwenhoek Hospital, Plesmanlaan 121, 1066 CX Amsterdam, The Netherlands; ^2^Biomedical Photonic Imaging Group, MIRA Institute, University of Twente, Post Box 217, 7500 AE Enschede, The Netherlands; ^3^Melanoma Institute Australia, 40 Rocklands Road, North Sydney, NSW 2060, Australia; ^4^Fujifilm Visualsonics Inc., Post Box 66, 3080 Yonge Street 6100, Toronto, ON, Canada; ^5^Department of Pathology, The Netherlands Cancer Institute, Antoni van Leeuwenhoek Hospital, Plesmanlaan 121, 1066 CX Amsterdam, The Netherlands; ^6^Nanobiophysics Group, MIRA Institute, University of Twente, Post Box 217, 7500 AE Enschede, The Netherlands

## Abstract

*Objective*. Sentinel node biopsy in patients with cutaneous melanoma improves staging, provides prognostic information, and leads to an increased survival in node-positive patients. However, frozen section analysis of the sentinel node is not reliable and definitive histopathology evaluation requires days, preventing intraoperative decision-making and immediate therapy. Photoacoustic imaging can evaluate intact lymph nodes, but specificity can be hampered by other absorbers such as hemoglobin. Near infrared multispectral photoacoustic imaging is a new approach that has the potential to selectively detect melanin. The purpose of the present study is to examine the potential of multispectral photoacoustic imaging to identify melanoma metastasis in human lymph nodes. *Methods*. Three metastatic and nine benign lymph nodes from eight melanoma patients were scanned *ex vivo* using a Vevo LAZR^©^ multispectral photoacoustic imager and were spectrally analyzed per pixel. The results were compared to histopathology as gold standard. *Results*. The nodal volume could be scanned within 20 minutes. An unmixing procedure was proposed to identify melanoma metastases with multispectral photoacoustic imaging. Ultrasound overlay enabled anatomical correlation. The penetration depth of the photoacoustic signal was up to 2 cm. *Conclusion*. Multispectral three-dimensional photoacoustic imaging allowed for selective identification of melanoma metastases in human lymph nodes.

## 1. Introduction

Sentinel node biopsy in patients with melanoma improves staging and guides the subsequent management of the nodal basin. The tumor status of the sentinel node is the most important prognostic factor. Sentinel node biopsy plus complete node dissection improves ten-year survival in node-positive patients [1]. Routine histopathology evaluation requires several days, preventing intraoperative decision making and immediate lymph node dissection. Less time consuming procedures like imprint cytology and frozen section analysis are characterized by a low sensitivity. Intraoperative imprint cytology and frozen section show a sensitivity of approximately 30 and 50%, respectively [2, 3]. Also noninvasive imaging techniques like ultrasound, computed tomography (CT), magnetic resonance imaging (MRI), and positron emission tomography (PET) lack the sensitivity to reliably detect micrometastases [4].

Photoacoustic imaging is a hybrid imaging technique, combining high-resolution ultrasound with molecular specific optical excitation. The method is based on optical absorption of pulsed light by specific tissue. The absorbed optical energy is converted to heat, which generates ultrasound waves by thermal expansion. Detection of these light-induced ultrasound waves allows recovering of the location of the ultrasound sources, which are the tissue structures that absorb the light. In general, structures that absorb more light will cause a stronger ultrasound signal. The absorption of light will differ at various wavelengths, as a molecular property. A multispectral approach makes use of this property to selectively visualize metastases.

Melanin is a light absorbing substance and photoacoustic imaging has been shown to detect melanin in lymph node metastases from melanoma using a single wavelength [5, 6]. Differentiation between blood and melanoma proved to be difficult because both are strong optical absorbers and therefore create a clear photoacoustic signal.

Wang used two-wavelength photoacoustic imaging to visualize thin superficial melanomas in mice using both red and infrared light [7]. Although the differentiation between melanin and blood was possible, the use of red light limits the penetration depth of this approach. For example, in this study the visualized melanoma was 0.3 mm thick and located at 0.32 mm under the skin of a nude mouse. Human lymph nodes are located at a depth of several centimeters in the human body. Near infrared light penetrates deeper into the tissue. Near infrared multispectral photoacoustic imaging may be able to selectively visualize melanin and blood at centimeters depth in intact human lymph nodes.

This study explores the potential of this new approach to identify melanoma metastasis* ex vivo* in human lymph nodes. A specific purpose was to correlate spectral images with known reference spectra to differentiate melanoma metastases from blood related artifacts in intact human lymph nodes. The other purpose was to investigate the value of a hand-held photoacoustic-ultrasound system capable of 3D imaging.

## 2. Materials and Methods

### 2.1. Photoacoustic Setup

The data was acquired using the Vevo LAZR^©^ Photoacoustic Imaging System (FUJIFILM VisualSonics Inc., Toronto), a hybrid photoacoustic-ultrasound reflection mode imager with a 21 MHz (13–24 MHz) linear array transducer. Fiber optic bundles near the surface of the transducer coupled to a 20 Hz tunable laser are able to deliver 20 mJ/cm^2^ in the wavelength range of 680–970 nm. For three-dimensional image acquisition, the transducer was automatically moved over the lymph node by a stepping motor with a step size of 0.2 mm. Both ultrasound and photoacoustic images were exported as 16 bit greyscale images to MATLAB (version 7.14; Mathworks, Natick, MA, USA) for image processing.

### 2.2. Phantom

In order to verify whether multispectral photoacoustic imaging is able to differentiate the chromophores blood from melanin, a phantom was developed. This was made of absorbing and scattering agar gel (2% in water) mimicking the optical properties of soft tissue. Embedded inside the phantom at 4 ± 1 mm depth were two 2% agar cylinders (diameter 2 mm, height 6 mm). One cylinder contained bovine hemoglobin (Hb) (Sigma-Aldrich, Zwijndrecht, the Netherlands) in a concentration of 15 g/L. The second cylinder contained B-16 melanin producing melanoma cells (2 × 10^6^ cells/mm^3^). The background consisted of 2% agar completely covering the 6 mm high cylinders with a cover on the top of 4 ± 1 mm. Multispectral volume scanning was performed using five distinct illumination wavelengths between 680 and 840 nm with 40 nm intervals. After image acquisition, the photoacoustic spectrum was obtained by selecting a 3D region of interest in each inclusion and calculating the mean values with standard deviations.

### 2.3. Human Lymph Nodes

Twelve human lymph nodes from eight melanoma patients undergoing lymphadenectomy were obtained from the surgical specimen. The experimental protocol was performed according to the Dutch guidelines for clinical research and patient's informed consent was acquired prior to surgery. The lymph nodes were stored in phosphate buffered saline before and during imaging. Subsequently, histopathology examination was performed using two or more slides stained with hematoxylin and eosin and biomarkers (S100, HMB-45, Melan-A) when needed.

In order to obtain accurate reference spectra for both blood and melanin, multispectral images were acquired for each node. Five wavelengths were selected: 3 wavelengths (700 nm, 800 nm, and 820 nm) covered the near infrared range. Two additional near infrared wavelengths (732 nm and 757 nm) were selected based on the absorption spectrum of oxy- and deoxyhemoglobin; both show an increase between 732 nm and 757 nm, whereas the absorption spectrum of melanin shows a slight decrease. In addition, at least one hyperspectral slice was acquired of every node using 101 colors of light, ranging from 700 to 900 nm with intervals of 2 nm. The photoacoustic reference spectrum of blood was obtained by selecting the vessels on the images of benign lymph nodes. The selected regions were also examined with high-resolution ultrasound and histology. In the metastatic nodes, ultrasound and histological slides were used to select an area in the photoacoustic dataset with a high melanin concentration as reference for melanin. Calculation of both a mean and a standard deviation for every measured wavelength resulted in reference spectra for both chromophores.

### 2.4. Unmixing Algorithm

The raw photoacoustic data were first filtered with a median filter. A two-dimensional median filter (block size 5 × 5 pixels in the image plane: approximately 110 × 110 *μ*m) was chosen because of the noncubical voxel dimensions: 22 × 22 × 200 *μ*m (depth × width × slice thickness). Variations in local fluence are expected, as light is absorbed and scattered while travelling through the heterogeneous tissue. Variations in light distribution were corrected by area under the curve normalization, that is, divided by the integral over the wavelength range. This correction aims to preserve the spectral characteristics, while the influence of absolute light intensity is reduced. The normalized measured signal was then compared to the normalized reference spectra of both blood and melanoma per image voxel. The reference spectra were based on 1168 voxels for blood and 1349 for melanin, selected per wavelength from a 3D region of interest.

In order to calculate the resemblance of the measured spectrum and the reference spectra, a statistical *t*-test was performed. The *P* value (ranging 0-1) was displayed in a resulting image. The *P* value was calculated for both blood and melanin for each of the five measured wavelengths. A value close to 1 indicates high resemblance; a value closer to 0 implies an increasing discrepancy with the reference. The five *P* values are combined by multiplication, resulting in a single matching score of the voxel per reference. Voxels exactly matching the reference at all five wavelengths received an intensity of 1^5^ = 1, while voxels with two standard deviations scored 0.05^5^ = 3 × 10^−7^. Matching pixels light up on the resulting image, while nonmatching pixels remain dark. These grayscale images are ultimately fused with the US images, with red representing blood and green representing melanin.

## 3. Results

### 3.1. Phantom

**Figure 1:**
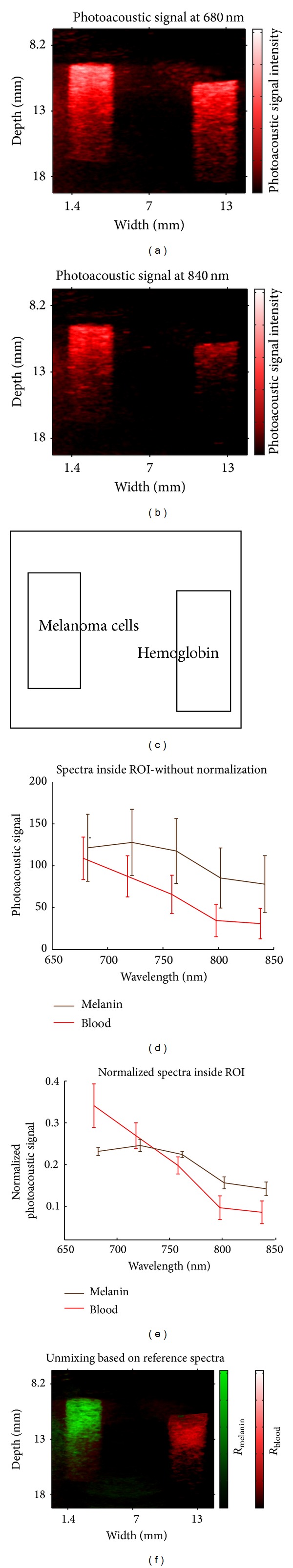
(a) Photoacoustic image of phantom after illumination with 680 nm light. (b) Photoacoustic image of phantom after illumination with 840 nm light. (c) Relative position of the inclusions in (a), (b), and (f). (d) Measured spectra for the two regions indicated in (c) for five wavelengths before normalization. The curves represent average and error bars indicate standard deviation. (e) Reference spectra after per-pixel normalization. The curves represent average and the error bars indicate standard deviation. (f) Resemblance score for blood (*R*_blood_) and melanin (*R*_melanin_) expressed as pseudocolors, hemoglobin in red and melanin in green.

Figure 1 shows the photoacoustic intensity maps of the phantom at 680 nm (Figure 1(a)) and 840 nm (Figure 1(b)). The position of the reference inclusions is schematically visualized in Figure 1(c). Both inclusions have a signal above background, especially at the superficial part. The signal intensity inside the inclusions decreases with depth as light fluence decreases deeper inside the phantom. Examination of the photoacoustic images alone does not allow for discrimination between blood and melanin. The information for discrimination lies in combination of multiple images, acquired at different wavelengths of  light. The spectra derived from the multispectral images show different patterns for blood and melanin (Figures 1(d) and 1(e)). The effect of area under the curve (AUC) normalization is characterized in the difference between the two curves. The result of the recognition algorithm shows that the two inclusions can be distinguished (Figure 1(f)). Some matching pixels were found outside the inclusions, indicating some margin of error that could result in false positive or false negative pixel classifications.

### 3.2. Human Lymph Nodes

**Figure 2:**
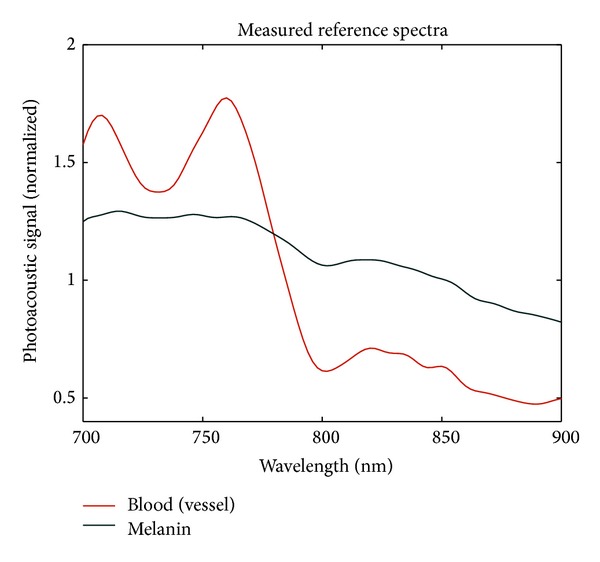
Normalized measured reference spectra for blood and melanin. The spectral differences between blood and melanoma are best reflected in the gradual slope of melanoma compared to the increase of blood in the wavelength range between 732 and 756 nm.

An overview of the measured nodes is provided in Table 1. Three of the twelve lymph nodes contained melanoma metastases according to histopathological examination, one of which was amelanotic. The reference spectrum of blood was based on over 8000 pixels of blood vessels in four tumor-negative lymph nodes from four different patients (Figure 2). The reference spectrum of melanin was based on over 2200 pixels of two tumor-positive lymph nodes from two patients. The spectral differences between blood and melanoma are best reflected in the gradual slope of the photoacoustic signal from melanoma compared to the increase in photoacoustic signal from blood in the wavelength range between 732 and 0756 nm in Figure 2.

**Figure 3:**
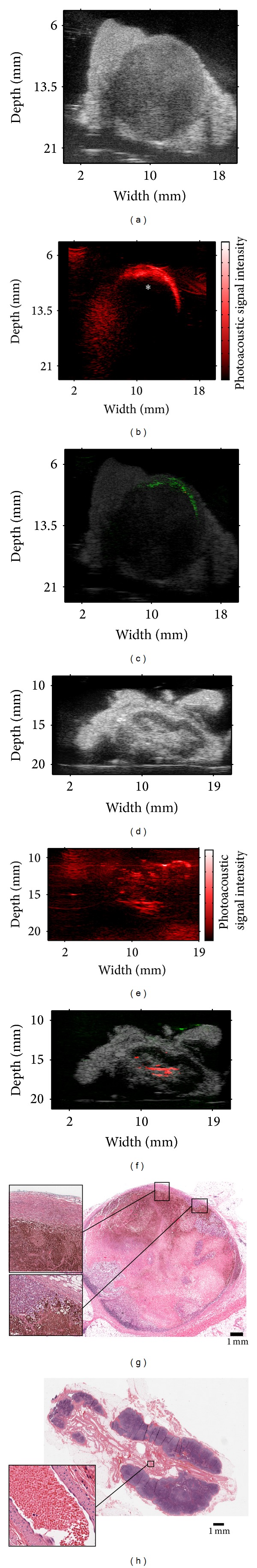
Images of two human nodes. The first row shows images of a metastatic node (node number 2 from Table 1); the second row shows images of a benign node (node number 3 from Table 1). Three columns represent, respectively, high-resolution ultrasound, photoacoustic signal, and the calculated resemblance. The third row shows the pathology slides of the metastatic node (g) and the normal node (h). Absence of photoacoustic signal deeper in the malignant node (as indicated by * in (b)) seems to be caused by the strong absorption by the melanin in the superficial area of the node. The green area in (c) corresponds to the superficial layer of the dark area in (g). The red structure in (f) corresponds to the vessels in the hilum of the benign lymph node (h).

The ultrasound image of a tumor positive lymph node, node number 2 in Table 1, (Figure 3(a)) visualizes the node as a round structure, surrounded by hyperechogenic fatty tissue. The corresponding photoacoustic image shows strong signal at the surface of the node, nearby the detector, while lack of signal can be noticed at a larger distance from the surface. The strong optical absorption of the melanin deposits limited the light penetration to 2-3 mm. The result of the spectral unmixing procedure (Figure 3(c)) shows a green strip overlaying the ultrasound image. The green represents image elements spectrally according to melanin. In this figure, almost no elements were found with a spectral response corresponding to blood. This is in accordance with the histological slide (Figure 3(g)) in which melanin is found in the upper part of the node (dark brown) and no larger vessels are seen inside the nodal capsule.

A representative benign node, node number 3 in Table 1, shows larger vessels entering the hilum of the node (Figures 3(d)–3(f) and 3(h)). The ultrasound (Figure 3(d)) visualizes a bean shaped node with a brighter hilum surrounded by bright fatty tissue. The photoacoustic image (Figure 3(e)) shows vessel shaped structures at 16 mm depth and signal at the surface of the fatty tissue. The vessel shaped structures are recognized as blood in the algorithm-result imaging; the signal at the surface of the fatty tissue does not resemble the reference of blood nor melanin. Histopathology slides (Figure 3(h)) confirm the location of the blood vessels within the hilum of the node. Within 20 minutes, total volume imaging proved possible for the benign lymph nodes, where penetration was sufficient to examine the entire nodes, which were up to 2 cm in diameter (Figure 3).

**Figure 4:**
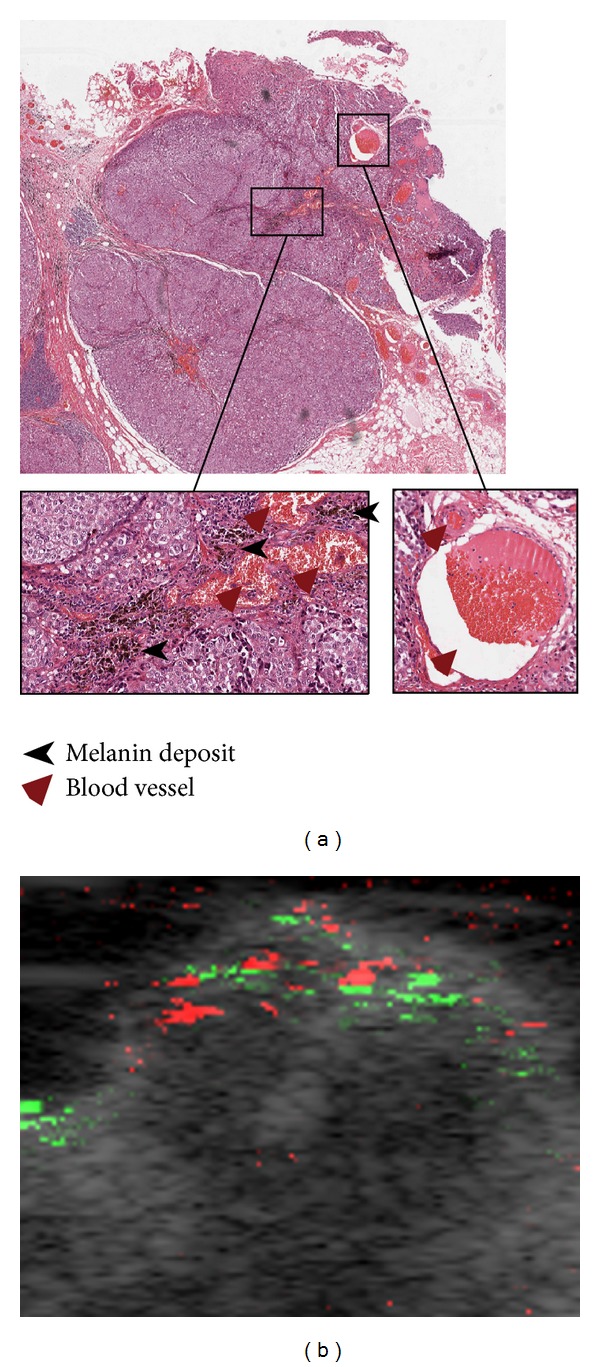
Pathology slide (a) and photoacoustic image (b) image of a second tumor-positive lymph node (node 11 from Table 1). This node has more blood vessels and less melanin deposits compared to the node in Figure 3. Blood vessels and melanin in the upper part of the node are more intertwined.

Tumor-positive nodes have different melanin distribution, which may result in different photoacoustic signal distribution. In Figure 4, the photoacoustic signal of node number 11 from Table 1, containing tumor metastasis, is compared with its corresponding histopathology slide. Compared to the first tumor-positive lymph node (Figure 3), the melanin distribution is more scattered throughout the node (Figure 4(a)) resulting in a more diffuse distribution of green photoacoustic pixels (Figure 4(b)). Also the number of blood vessels seems higher in this node which is reflected in the corresponding photoacoustic images (Figures 4(b) and 3(c)). Direct comparison of the photoacoustic images and the pathology slides is, however, hampered by the handling of the nodes in between.

## 4. Discussion

Earlier studies showed that photoacoustic imaging can detect melanin in lymph nodes, but the specificity was low as the signal from melanin could not be differentiated from blood. The results of this feasibility study show that it is possible to distinguish the photoacoustic signals of blood and melanin both in phantoms and in resected human lymph nodes. The implemented unmixing procedure allows discrimination between melanin and blood. Because the technique is based on spectral comparison, one should keep in mind that the accuracy of unmixing is strongly tied to the reference spectra. The presented reference spectra show similarity with known optical absorption spectra for hemoglobin (oxyhemoglobin + deoxyhemoglobin) and eumelanin [8, 9]. The gradual slope in the spectrum of melanin and the increase between 732 nm and 756 nm for hemoglobin is characteristic in both optical absorption and the reference spectra. However, the reference spectra should encompass the response in a variety of situations, which requires a multitude of human samples to be scanned in further studies.

Specific detection of melanin can be useful in intraoperative photoacoustic imaging of an excised sentinel node. This would permit an immediate node dissection if metastatic foci are demonstrated and would obviate the need for a second operation. Perhaps the sentinel node could even be analyzed* in vivo*. A penetration depth of 2 cm in the absence of melanin deposits enables the analysis of the entire nodal volume. This is in contrast with the conventional pathology evaluation that samples less than 0.1% of a node.

When present, melanin absorbs most of the light and limits the depth of photoacoustics imaging under these circumstances. For clinical application this may be of limited importance, since the extent of tumor involvement per node is presently of less relevance. Also the presence of a blue dye, generally used for sentinel node detection, may alter the penetration depth of optical imaging. However, the impact on melanoma detection will be limited since the optical absorption of blue dye in the infrared region is limited and the optical absorption spectrum of the dye is clearly distinctive from both blood and melanin.

A false positive test result may be realistic because normal naevus cells are present in one-third of the skin draining lymph nodes. Additional research should reveal whether adequate reference spectra for these kinds of conditions can still be defined.

As photoacoustic imaging relies on optical absorption, the 1.8% to 8.1% amelanotic melanomas may challenge the sensitivity of the procedure [10]. An occasional false negative procedure will not have major detrimental consequences, because a strong point of photoacoustic scanning is that histological and immunohistochemical analysis afterwards remains possible. Therefore, a false negative procedure does permit a node dissection, albeit at a somewhat later date. Recent reports describing molecular based staging methods do not allow for this option, because these techniques destroy the tissue during the procedure [11, 12].

For* ex vivo* robust real time sentinel lymph node analysis, further research should initial focus on the accuracy of photoacoustic imaging for small lymph node metastases.

## 5. Conclusion

A method is proposed to detect melanoma metastases in human lymph nodes using multispectral photoacoustic imaging. The method was conducted on a set of lymph nodes containing tumor positive and tumor negative nodes. Separation of important optical absorbers like blood and melanin proved possible based on the spectral information. Lymph nodes could be analyzed in image planes and also as entire nodal volumes (3D).

In hybrid photoacoustic imaging, the high resolution ultrasound imaging can be used for orientation, as well as for diagnosis, as in conventional ultrasound. The two techniques are synergetic as they are based on different imaging contrasts; ultrasound is based on acoustic impedance which is sensitive to anatomy and photoacoustics on light absorption which is sensitive to molecular properties. The reflective mode hand-held system proved capable of 3D imaging of the entire lymph node. Further research should be directed towards the robustness of this technology for the detection of small melanoma lesions within human lymph nodes and its specificity.

## Figures and Tables

**Table 1:** Patient characteristics.

Patient	Age	Gender	Node	Histology	Node longest diameter (cm)	Used as reference
1	37	Male	1	Benign	0.5	
2	65	Female	2	Malignant	1.4	Yes
3	24	Female	3	Benign	1.0	Yes
4	62	Male	4	Benign	0.9	
			5	Benign	1.1	Yes
5	79	Female	6	Malignant∗	1.2	
6	62	Female	7	Benign	0.6	
			8	Benign	1.5	Yes
7	38	Male	9	Benign	1.2	
			10	Benign	0.8	
8	25	Female	11	Malignant	1.2	Yes
			12	Benign	1.8	Yes

∗Amelanotic melanoma.
